# Draft Genome Sequence of Halotolerant Polycyclic Aromatic Hydrocarbon-Degrading *Pseudomonas bauzanensis* Strain W13Z2

**DOI:** 10.1128/genomeA.01049-14

**Published:** 2014-10-16

**Authors:** Xinxin Wang, Decai Jin, Lisha Zhou, Liang Wu, Lin Qi, Chen Li, Wei An, Yu Chen

**Affiliations:** a School of Environmental Science and Engineering, Tianjin University, Tianjin, China; b China Offshore Environmental Service Co. Ltd., Tianjin, China; c College of Resources and Environment, University of Chinese Academy of Sciences, Beijing, China; d Research Center for Eco-Environmental Sciences, Chinese Academy of Sciences, Beijing, China; e Shenzhen Key Laboratory of Environmental Microbial Genomics and Application, BGI, Shenzhen, China; f College of Environmental Science and Engineering, Ocean University of China, Qingdao, China; g School of Chemical Engineering and Technology, Tianjin University, Tianjin, China

## Abstract

*Pseudomonas bauzanensis* W13Z2 is a halotolerant polycyclic aromatic hydrocarbon (PAH)-degrading bacterium isolated from petroleum-contaminated drill cuttings in the Bohai Sea. Here, we report the 8.6-Mb draft genome sequence of this strain, which will provide insights into the diversity of *Pseudomonas* and the mechanism of PAHs degradation in drill cuttings.

## GENOME ANNOUNCEMENT

Microbial populations in drill cuttings have been well documented (1, 2). However, little is known about polycyclic aromatic hydrocarbon (PAH)-degrading bacteria in such an environment. *Pseudomonas bauzanensis* W13Z2, which can degrade phenanthrene and pyrene with 5% NaCl, was isolated from petroleum-contaminated drill cuttings in the Bohai Sea of China. However, genomic information about *P. bauzanensis* is still unknown, which limits understanding of the mechanism of PAH degradation in drill cuttings. Here, the draft genome sequence of *P. bauzanensis* W13Z2 is presented for the first time.

Genomic DNA was extracted and sequenced using an Illumina HiSeq 2000 platform. The shotgun sequencing produced 14,417,292 paired-end reads with an average insert size of 300 bp (yielding approximately 170-fold coverage), which were filtered by NGS QC toolkit v 2.3 (3). Filtered reads were assembled, scaffolded, gap filled, and validated using SOAPdenovo v. 2.04 (4), SSPACE v. 2.0 (5), GapFiller v. 1.10 (6), and bwa v. 0.7.4 (7). Final assembly consisted of 69 contigs with an *N*_50_ length of 289,896 bp and the largest length of 1,692,974 bp. The genome sequence was annotated using the NCBI Prokaryotic Genome Automatic Annotation Pipeline (PGAAP) (http://www.ncbi.nlm.nih.gov/genome/annotation_prok/).

The genome consists of 8.6 Mb, with a G+C content of 61.8%. A total of 7,839 coding sequences (CDS), 139 pseudogenes, 97 tRNA genes, and 2 noncoding RNA (ncRNA) and 11 rRNA genes were identified. Of the CDS, 76.5% were assigned to clusters of orthologous groups (COGs) with amino acid transport and metabolism as the most abundant class, and 43.3% can be annotated into 2,479 KEGG orthologous groups by using KAAS (8), involving 218 metabolic pathways. A total of 209 tandem repeats were detected by Tandem Repeats Finder v. 4.07 (9). The IS*5* family dominated the insertion sequence (IS) elements as revealed by ISFinder (10). A total of 695 potentially secreted proteins were identified by SignalP v. 4.0 (11). One clustered regularly interspaced short palindromic repeat (CRISPR) element with 20 spacers was identified by CRISPRFinder (12). Average nucleotide identity (ANI) analysis (13) revealed that *P. bauzanensis* W13Z2 is phylogenetically related to *Pseudomonas aeruginosa* PAO1 (70.5%) (14), *P. brassicacearum* NFM421 (69.55%) (15), *P. denitrificans* ATCC 13867 (70.7%) (16), *P. entomophila* L48 (70.2%) (17), *P. mendocina* NK01 (70.5%) (18), *P. monteilii* SB3078 (70.0%) (19), *P. poae* RE*1/1/14 (69.7%) (20), *P. putida* KT2440 (69.8%) (21), *P. resinovorans* NBRC106553 (70.8%) (22), *P. stutzeri* A1501 (70.8%) (23), and *P. syringae* pv. tomato DC3000 (69.2%) (24).

Thirteen genes responsible for the degradation of alkanes and PAHs were identified, including 1 alkane 1-monooxygenase gene, 5 catechol 1,2-dioxygenase genes, 2 benzene 1,2-dioxygenase genes, and 5 naphthalene 1,2-dioxygenase genes. Moreover, 9 genes were identified as involved in compatible solute synthesis and uptake, including 3 betaine-aldehyde dehydrogenase, 5 glycine/betaine ABC transporter genes, and 1 ectoine synthase genes. Copper-, mercury-, and tellurium-resistant genes were detected, which may enhance the resistance to heavy metal. Eleven cold shock protein genes were detected, which are helpful for the survival in seawater at low temperatures.

### Nucleotide sequence accession number.

The draft genome sequence of *P. bauzanensis* W13Z2 has been deposited in GenBank under the accession number JFHS00000000. The version described in this paper is the first version.

## References

[1] Benka-Coker MO Olumagin A. 1995. Waste drilling-fluid-utilising microorganisms in a tropical mangrove swamp oil field location. Bioresour. Technol. 53:211–215. 10.1016/0960-8524(95)00055-1

[2] Struchtemeyer CG Davis JP Elshahed MS. 2011. Influence of the drilling mud formulation process on the bacterial communities in thermogenic natural gas wells of the Barnett shale. Appl. Environ. Microbiol. 77:4744–4753. 10.1128/AEM.00233-11 21602366 PMC3147393

[3] Patel RK Jain M. 2012. NGS QC Toolkit: a toolkit for quality control of next generation sequencing data. PLoS One 7:e30619. 10.1371/journal.pone.0030619 22312429 PMC3270013

[4] Luo R Liu B Xie Y Li Z Huang W Yuan J He G Chen Y Pan Q Liu Y Tang J Wu G Zhang H Shi Y Liu Y Yu C Wang B Lu Y Han C Cheung DW Yiu SM Peng S Xiaoqian Z Liu G Liao X Li Y Yang H Wang J Lam TW Wang J. 2012. SOAPdenovo2: an empirically improved memory-efficient short-read *de novo* assembler. Gigascience 1:18. 10.1186/2047-217X-1-18 23587118 PMC3626529

[5] Boetzer M Henkel CV Jansen HJ Butler D Pirovano W. 2011. Scaffolding pre-assembled contigs using SSPACE. Bioinformatics 27:578–579. 10.1093/bioinformatics/btq683 21149342

[6] Boetzer M Pirovano W. 2012. Toward almost closed genomes with GapFiller. Genome Biol. 13:R56. 10.1186/gb-2012-13-6-r56 22731987 PMC3446322

[7] Li H Durbin R. 2009. Fast and accurate short read alignment with Burrows-Wheeler transform. Bioinformatics 25:1754–1760. 10.1093/bioinformatics/btp324 19451168 PMC2705234

[8] Moriya Y Itoh M Okuda S Yoshizawa AC Kanehisa M. 2007. KAAS: an automatic genome annotation and pathway reconstruction server. Nucleic Acids Res. 35:W182–W185. 10.1093/nar/gkm321 17526522 PMC1933193

[9] Benson G. 1999. Tandem repeats finder: a program to analyze DNA sequences. Nucleic Acids Res. 27:573–580. 10.1093/nar/27.2.573 9862982 PMC148217

[10] Siguier P Perochon J Lestrade L Mahillon J Chandler M. 2006. ISfinder: the reference centre for bacterial insertion sequences. Nucleic Acids Res. 34:D32–D36. 10.1093/nar/gkj014 16381877 PMC1347377

[11] Petersen TN Brunak S von Heijne G Nielsen H. 2011. SignalP 4.0: discriminating signal peptides from transmembrane regions. Nat. Methods 8:785–786. 10.1038/nmeth.1701 21959131

[12] Grissa I Vergnaud G Pourcel C. 2007. CRISPRFinder: a web tool to identify clustered regularly interspaced short palindromic repeats. Nucleic Acids Res. 35:W52–W57. 10.1093/nar/gkm360 17537822 PMC1933234

[13] Richter M Rosselló-Móra R. 2009. Shifting the genomic gold standard for the prokaryotic species definition. Proc. Natl. Acad. Sci. U. S. A. 106:19126–19131. 10.1073/pnas.0906412106 19855009 PMC2776425

[14] Stover CK Pham XQ Erwin AL Mizoguchi SD Warrener P Hickey MJ Brinkman FS Hufnagle WO Kowalik DJ Lagrou M Garber RL Goltry L Tolentino E Westbrock-Wadman S Yuan Y Brody LL Coulter SN Folger KR Kas A Larbig K Lim R Smith K Spencer D Wong GK Wu Z Paulsen IT Reizer J Saier MH Hancock RE Lory S Olson MV. 2000. Complete genome sequence of *Pseudomonas aeruginosa* PAO1, an opportunistic pathogen. Nature 406:959–964. 10.1038/35023079 10984043

[15] Ortet P Barakat M Lalaouna D Fochesato S Barbe V Vacherie B Santaella C Heulin T Achouak W. 2011. Complete genome sequence of a beneficial plant root-associated bacterium, *Pseudomonas brassicacearum*. J. Bacteriol. 193:3146. 10.1128/JB.00411-11 21515771 PMC3133198

[16] Ainala SK Somasundar A Park S. 2013. Complete genome sequence of *Pseudomonas denitrificans* ATCC 13867. Genome Announc. 1(3):e00257-13. 10.1128/genomeA.00257-13 23723394 PMC3668002

[17] Vodovar N Vallenet D Cruveiller S Rouy Z Barbe V Acosta C Cattolico L Jubin C Lajus A Segurens B Vacherie B Wincker P Weissenbach J Lemaitre B Médigue C Boccard F. 2006. Complete genome sequence of the entomopathogenic and metabolically versatile soil bacterium *Pseudomonas entomophila*. Nat. Biotechnol. 24:673–679. 10.1038/nbt1212 16699499

[18] Guo W Wang Y Song C Yang C Li Q Li B Su W Sun X Song D Yang X Wang S. 2011. Complete genome of *Pseudomonas mendocina* NK-01, which synthesizes medium-chain-length polyhydroxyalkanoates and alginate oligosaccharides. J. Bacteriol. 193:3413–3414. 10.1128/JB.05068-11 21551299 PMC3133264

[19] Dueholm MS Albertsen M D’Imperio S Tale VP Lewis D Nielsen PH Nielsen JL. 2014. Complete genome sequences of *Pseudomonas monteilii* SB3078 and SB3101, two benzene-, toluene-, and ethylbenzene-degrading bacteria used for bioaugmentation. Genome Announc. 2(3):e00524-14. 10.1128/genomeA.00524-14 24874689 PMC4038894

[20] Müller H Zachow C Alavi M Tilcher R Krempl PM Thallinger GG Berg G. 2013. Complete genome sequence of the sugar beet endophyte *Pseudomonas poae* Re*1-1-14, a disease-suppressive bacterium. Genome Announc. 1(2):e00020-13. 10.1128/genomeA.00020-13 PMC3593332 23516179

[21] Nelson KE Weinel C Paulsen IT Dodson RJ Hilbert H Martins dos Santos VA Fouts DE Gill SR Pop M Holmes M Brinkac L Beanan M DeBoy RT Daugherty S Kolonay J Madupu R Nelson W White O Peterson J Khouri H Hance I Chris Lee P Holtzapple E Scanlan D Tran K Moazzez A Utterback T Rizzo M Lee K Kosack D Moestl D Wedler H Lauber J Stjepandic D Hoheisel J Straetz M Heim S Kiewitz C Eisen JA Timmis KN Düsterhöft A Tümmler B Fraser CM. 2002. Complete genome sequence and comparative analysis of the metabolically versatile *Pseudomonas putida* KT2440. Environ. Microbiol. 4:799–808. 10.1046/j.1462-2920.2002.00366.x 12534463

[22] Shintani M Hosoyama A Ohji S Tsuchikane K Takarada H Yamazoe A Fujita N Nojiri H. 2013. Complete genome sequence of the carbazole degrader *Pseudomonas resinovorans* strain CA10 (NBRC 106553). Genome Announc. 1(4):e00488-13. 10.1128/genomeA.00488-13 23887915 PMC3735057

[23] Yan Y Yang J Dou Y Chen M Ping S Peng J Lu W Zhang W Yao Z Li H Liu W He S Geng L Zhang X Yang F Yu H Zhan Y Li D Lin Z Wang Y Elmerich C Lin M Jin Q. 2008. Nitrogen fixation island and rhizosphere competence traits in the genome of root-associated *Pseudomonas stutzeri* A1501. Proc. Natl. Acad. Sci. U. S. A. 105:7564–7569. 10.1073/pnas.0801093105 18495935 PMC2396677

[24] Buell CR Joardar V Lindeberg M Selengut J Paulsen IT Gwinn ML Dodson RJ Deboy RT Durkin AS Kolonay JF Madupu R Daugherty S Brinkac L Beanan MJ Haft DH Nelson WC Davidsen T Zafar N Zhou L Liu J Yuan Q Khouri H Fedorova N Tran B Russell D Berry K Utterback T Van Aken SE Feldblyum TV D’Ascenzo M Deng WL Ramos AR Alfano JR Cartinhour S Chatterjee AK Delaney TP Lazarowitz SG Martin GB Schneider DJ Tang X Bender CL White O Fraser CM Collmer A. 2003. The complete genome sequence of the Arabidopsis and tomato pathogen *Pseudomonas syringae* pv. tomato DC3000. Proc. Natl. Acad. Sci. U. S. A. 100:10181–10186. 10.1073/pnas.1731982100 12928499 PMC193536

